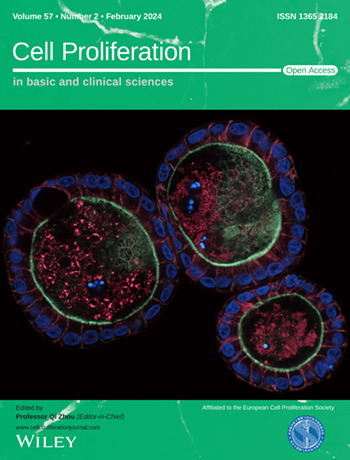# Additional Cover

**DOI:** 10.1111/cpr.13616

**Published:** 2024-02-07

**Authors:** Georg Csukovich, Maximilian Wagner, Ingrid Walter, Stefanie Burger, Waltraud Tschulenk, Ralf Steinborn, Barbara Pratscher, Iwan Anton Burgener

## Abstract

The cover image is based on the Original Article *Polarity reversal of canine intestinal organoids reduces proliferation and increases cell death* by Georg Csukovich et al., https://doi.org/10.1111/cpr.13544. Image Credit: Georg Csukovich.